# Telomere Shortening Sensitizes Cancer Cells to Selected Cytotoxic Agents: *In Vitro* and *In Vivo* Studies and Putative Mechanisms

**DOI:** 10.1371/journal.pone.0009132

**Published:** 2010-02-09

**Authors:** Orit Uziel, Einat Beery, Vladimir Dronichev, Katty Samocha, Sergei Gryaznov, Lola Weiss, Shimon Slavin, Michal Kushnir, Yardena Nordenberg, Claudette Rabinowitz, Baruch Rinkevich, Tania Zehavi, Meir Lahav

**Affiliations:** 1 Beilinson Hospital, Rabin Medical Center, Felsenstein Medical Research Center, Petah-Tikva, Sackler School of Medicine, Tel-Aviv University, Tel-Aviv, Israel; 2 Geron Corporation, Menlo Park, California, United States of America; 3 Department of Bone Marrow Transplantation and Cancer Immunotherapy, Hadassah University Hospital, Jerusalem, Israel; 4 Rosetta Genomics Ltd, Rehovot, Israel; 5 National Institute of Oceanography, Haifa, Israel; 6 Department of Pathology, Meir Medical Center, Kfar-Saba, Israel; 7 Beilinson Hospital, Rabin Medical Center, Felsenstein Medical Research Center, Petah-Tikva, Department of Human Molecular Genetics and Biochemistry, Sackler School of Medicine, Tel-Aviv University, Tel-Aviv, Israel; Roswell Park Cancer Institute, United States of America

## Abstract

**Background:**

Telomere/telomerase system has been recently recognized as an attractive target for anticancer therapy. Telomerase inhibition results in tumor regression and increased sensitivity to various cytotoxic drugs. However, it has not been fully established yet whether the mediator of these effects is telomerase inhibition *per se* or telomere shortening resulting from inhibition of telomerase activity. In addition, the characteristics and mechanisms of sensitization to cytotoxic drugs caused by telomerase inhibition has not been elucidated in a systematic manner.

**Methodology/Principal Findings:**

In this study we characterized the relative importance of telomerase inhibition versus telomere shortening in cancer cells. Sensitization of cancer cells to cytotoxic drugs was achieved by telomere shortening in a length dependent manner and not by telomerase inhibition *per se*. In our system this sensitization was related to the mechanism of action of the cytotoxic drug. In addition, telomere shortening affected also other cancer cell functions such as migration. Telomere shortening induced DNA damage whose repair was impaired after administration of cisplatinum while doxorubicin or vincristine did not affect the DNA repair. These findings were verified also in *in vivo* mouse model. The putative explanation underlying the phenotype induced by telomere shortening may be related to changes in expression of various microRNAs triggered by telomere shortening.

**Conclusions/Significance:**

To our best knowledge this is the first study characterizing the relative impact of telomerase inhibition and telomere shortening on several aspects of cancer cell phenotype, especially related to sensitivity to cytotoxic drugs and its putative mechanisms. The microRNA changes in cancer cells upon telomere shortening are novel information. These findings may facilitate the development of telomere based approaches in treatment of cancer.

## Introduction

Human telomeres are composed of single stranded TTAGGG repeats and corresponding duplexes of this hexanucleotide located at both ends of the linear chromosome. Together with their specific shelterin protein complex they provide stability to the whole genome, by masking chromosome ends from being treated by DNA repair mechanisms as double strand breaks [1]. Telomeres incrementally erode in most somatic cells upon each round of DNA replication, until they reach critical short length which eventually initiates a cessation of cell growth termed cellular senescence [2]. Cancer cells use the enzyme telomerase to circumvent telomere shortening, and thus achieve endless replicative potential. Telomerase is a unique reverse transcriptase ribonucleoprotein complex that maintains a steady state of telomere length by synthesizing TTAGGG repeats at the ends of chromosomes. It is highly active in more than 90% of all malignancies, and therefore considered a hallmark of cancer [3]. Telomerase is not expressed in most normal somatic cells but retains moderate activity in proliferative stem cells and to a higher extent in male germ line cells. Due to this specificity and essentiality to the limitless lifespan of cancer cells, telomerase is considered a valid and attractive anticancer target [4].

Indeed, numerous studies have shown that telomerase inhibition results in apoptosis of cancer cells, shrinkage of tumors in experimental animal models and enhanced sensitivity of tumor cells to various anticancer modalities [5]. However, it is not clear whether these ”beneficial biologically desirable” effects are the consequence of telomere shortening or telomerase inhibition *per se*. In addition, it has not been determined yet if the enhancement of sensitivity to cytotoxic drugs by telomerase inhibition/telomere shortening is dependent on the mechanism or class of the chemotherapeutic agent.

Most of the data point to shortening of telomeres below a critical limit as the most important target achieved by telomerase inhibition [6], [7], supporting the notion by which achievement of significant telomere shortening will result in anti cancer clinical effects. However, several studies implicate additional “extracurricular” activities of telomerase which are independent of telomere length regulation. For example, telomerase has been shown to possess antiapoptotic properties [8], [9]. In addition, its involvement in DNA damage response [10] or DNA protection by “capping” [11] was determined as well. Telomerase was also implicated in gene expression control irrespectively of telomere length and in contribution to growth of various types of benign [12]–[14] and malignant [15], [16] cells.

The aim of this study was to clarify the importance of telomerase inhibition *per* se versus the effect of telomere shortening in cancer cells and to evaluate the effect of these perturbations on the sensitivity of the cells to various cytotoxic drugs with different mechanisms of action. In addition, we aimed at depicting the mechanisms by which cells with shortened telomere length exhibit differential sensitivity to these drugs.

We have found that telomerase inhibition *per se* does not alter the sensitivity of several malignant cell lines to any of the drugs tested. Long term telomerase inhibition which resulted in telomere shortening sensitized the cells to cisplatinum, a DNA adducts forming agent and not to doxorubicin, a double strand breaks producing agent or to vincristine, which mode of action is not through direct DNA damage. These results were confirmed in an animal model employing nude mice with xenografts of pancreatic carcinoma cells which were exposed to telomerase inhibitor and cytotoxic drugs. Cells with shorter telomeres acquired DNA damage phenotype whose repair was impaired after cisplatinum only and expressed miRNA that are associated with growth arrest of cancer cells. These cells also presented slower migration compared to their wild type (WT) counterparts. We suggest that telomere shortening in cancer cells is associated with changes in miRNA expression and leads to impaired DNA repair after exposure to cisplatinum specifically.

## Materials and Methods

### Cell Lines

SK-N-MC (Ewing sarcoma) cell line was kindly provided by Dr Gad Lavie (Sheba Medical Center, Ramat-Gan, Israel). MCF-7 (breast carcinoma) and K562 (chronic myeloid leukemia) cells were maintained in RPMI 1640 supplemented with 10–15% heat-inactivated fetal calf serum (FCS), glutamine (2 mM), penicillin and streptomycin (Beit Haemek, Israel). Proliferation assays, apoptosis analyses and telomerase activity assays were performed on all cell lines. SK-N-MC line was chosen for further detailed analysis of various mechanisms related to the effect of telomerase inhibition on sensitivity to cisplatinum. The control cells were maintained in culture without telomerase inhibitor, GRN163, in parallel with the telomerase inhibited cells.

### Telomerase Inhibition

Cells were exposed twice a week to telomerase inhibitor GRN163, targeting the template region of RNA subunit of telomerase (hTR). The control cells were maintained in culture without telomerase inhibitor in parallel with the inhibited cells. For the *in vivo* inhibition of telomerase, mice were injected with GRN163L, a palmitoyl (C16) lipid-attached N3′-P5′ phosphoramidate version of GRN163. Both compounds were kindly provided by Geron Corporation (Menlo Park, CA, USA)

### Drug Treatment and *In Vitro* Experimental Protocol

All cell lines were exposed to the following drugs for three days prior to the proliferation, cell cycle and apoptosis analyses: Cisplatinum, a DNA adducts forming drug, doxorubicin, a double strand breaking agent and vincristine, which interferes with the formation of spindle microtubules, thus stops the separation of the duplicated chromosomes and prevents cell division. Concentration of the drugs is depicted in the results section.

To assess the effect of telomerase inhibition versus telomere shortening on the sensitivity of the cells to the cytotoxic drugs we devised four experimental conditions: 1. Telomerase was inhibited in the three cell lines for three days creating cells without telomerase activity and with intact (WT) telomeres. 2. Long term inhibition (from three to 16 months), creating cells without telomerase activity and shortened telomeres. 3. Withdrawal of the telomerase inhibitor in cells with shortened telomeres, creating cells with short telomeres and reconstituted telomerase activity. 4. As a control we used intact wild type cells. Telomere length and the IC50 of the three cytotoxic drugs were determined after 3 and 16 months in all three cell lines.

### Proliferation Assay

Adherent cells (1×10^4^ cells ml^−1^ SK-N-MC and MCF-7) were seeded in quadruplicate in 24-well plates. Various drugs were added at the following concentrations range: vincristine: 0–100 ng/ml, doxorubicin: 0–1000 ng/ml and cisplatinum: 0–10 µg/ml. After 3 days, proliferation was determined with the sulphorhodamine B assay [17]. The proliferation of the non-adherent K562 cells was determined by the WST-1 assay which follows the conversion of tetrazolium salt into formazan dye by mitochondrial enzymes according to the manufacturer's instructions (Roche, Germany) and as described previously [17].

### TRAP Assay

Cells (5×10^4^/ml) were plated in 24-well plates and incubated in the presence of GRN163 for 1–3 days. Each treatment was performed in duplicates. Subsequently, measurement of telomerase activity was performed by the PCR-based TRAP assay, using the TRAP_EZE_ telomerase detection kit (Intergene, NY, USA), according to the manufacturer's instructions and as previously described [17]. Briefly, cells were lysed with ice-cold CHAPS lysis buffer) for 30 min at 4°C and were subsequently centrifuged at 13,000 rpm for 30 min at 4°C. The supernatant was then collected and the protein concentration was determined by the Bradford assay (Bio-Rad Laboratories, CA, USA). Protein extracts (0.2 µg) were assayed for TRAP analysis. Each reaction was performed in a 50 µl reaction mixture containing 10×TRAP buffer, dNTP mix, TS primer, TRAP primer mix and Taq polymerase. Reactions were performed at 30°C for 30 min and were then subjected to PCR amplification for 30 cycles of 94°C, 58°C and 72°C for 30 s each, and were separated by electrophoresis on 12.5% polyacrylamide gels (Acryl/Bis 19∶1 solution). Gels were stained with SYBER Green nucleic acid gel stain (Amresco, Ohio, USA). Quantifications were performed using the Quantity-one software for Bio-Rad's Image analysis systems (Bio-Rad Laboratories, Israel). Telomerase activity was calculated according to the following formula: TPG = [(X−X_0_)/C]∶[(r−r_0_)/Cr*100], where TPG is the total product generated, X signifies each sample, C represents the 36 bp internal PCR control, r is the TSR8 quantification control.

### TRF Length

Terminal restriction fragment (TRF) length, corresponding to telomere length, was measured by a non-radioactive Southern blot technique. DNA samples were extracted from the cells by the Genomic DNA purification kit (Gentra, Minneapolis, MN, USA) according to the manufacturer's instructions, digested for 16 h by *HinfIII* and *RSAI* and separated on 0·8% agarose gels. After transfer to positively charged nylon membrane, samples were hybridized with digoxigenin-labelled probe (TTAGGG)_4_ (Roche Applied Science, Mannheim, Germany) for 16–18 h. The membranes were then exposed to chemiluminescence-sensitive films, and the average TRF lengths were calculated by the Versa Doc Imaging System, using Quantity One software (Bio-Rad Laboratories).

### Analyses of miR Signature

#### RNA isolation

RNA was isolated by using the EZ-RNA II commercial kit (Biological Industries, Beit Haemek, Israel) according to the manufacturer's instructions provided. In general, cells after relevant treatments were lysed in lysis buffer of the kit and stored at −70°C prior to RNA extraction which was done to all cells at once, prior to hybridization with the miRNA arrays.

#### MicroRNA microarray

Custom microRNA microarrays have been described previously [18]. Briefly, ∼900 DNA Oligonucleotide probes representing microRNA (Sanger database version 10 and additional microRNAs predicted and validated by Rosetta Genomics) were spotted in triplicates on coated microarray slides (Nexterion® Slide E. Schott, Mainz, Germany). Each RNA sample (15 µg of total RNA) was labeled by ligation of an RNA-linker, p-rCru-Cy/Dye (Dharmacon Lafayette, CO: Cy3 or Cy5) to the 3′ end. Slides were incubated with the labeled RNA for 12–16 hours at 42°C and then washed twice. Arrays were scanned at resolution of 10 µm and images were analyzed using SpotReader software (Niles Scientific Portola Valley, CA). Two types of positive controls were included in the experimental design: (i) synthetic small RNAs were spiked into each RNA sample before labeling to verify labeling efficiency and (ii) probes for abundant small RNAs were spotted to validate RNA quality. Microarray spots were combined and signals normalized as described previously [18].

#### Data analysis

The data included two samples: SK-N-MC cells with intact telomeres and SK-N-MC cells with shortened telomeres. A total of 170 microRNA probes had a signal that passed the minimal threshold of 300 in at least one of the samples. For each of those microRNA molecules we calculated the signal fold change between the samples with short telomeres and the sample with intact telomeres.

### Cell Cycle and Apoptosis Analyses

Cells (0.7–1×10^4^ ml^−1^) were cultured for 3 days. Floating and adherent cells were combined, washed with PBS and nuclei prepared from 5×10^5^ to 1×10^6^ cells for flow cytometric analysis using a detergent-trypsin method followed by staining with propidium iodide [19]. DNA content was analyzed by FACSCALIBUR (Becton Dickinson, San Jose, CA, USA), using ModFitLT cell cycle analysis software (Verity Software House Inc., Topsham, ME, USA). Apoptosis was assessed by FACS analysis by which cells in the pre-G1 stage of the cell cycle were defined as apoptotic.

### Detection of γH2AX Foci

Cells were plated on cover slides and exposed to the drugs for 24h, fixed and processed as previously described [20]. Briefly, cells were fixed with 4% paraformaldehide, washed three times with PBS, and permeabilized with 5% Triton X for 10 min. After three washes with PBS, cells were blocked by 10% normal donkey serum and 1% BSA, washed again with PBS and incubated with γH2AX antibody solution (1∶350, Upstate Biotechnology, NY, USA) for 16h at 4°C. The second antibody CY2 conjugated anti mouse (Jackson Immuno Research Laboratories, PA, USA) was added after three washed with PBS. Cover slides were stained with DAPI and mounting solution. γH2AX foci were visualized under the fluorescent microscope (Applied Spectral Imaging, Migdal Haemek, Israel). 50 nuclei were counted for each sample.

### The Comet Assay

DNA damage levels in cells with different telomere lengths and damages incurred after exposure to drugs were assessed by the comet assay, as described [21]. This assay follows the fragility level of the DNA by assessing DNA fragments migration (products of single and/or double strand breaks) from nucleus core to form comet shape when exposed to electrophoretic field. After staining with ethidium bromide, the extent of the comet can be monitored and quantified. Basically, cell suspension was mixed with 0.65% low-melting agarose and spread on a Star-frost microscope slide, pre-coated with 0.65% normal melting agarose. A third layer containing 0.65% low-melting agarose was placed on top and the cells were then lysed by immersing the slides overnight in a freshly prepared lysis solution (2.5 M NaCl, 100 mM EDTA, 10 mM Tris, 1% Triton X-100, 10% DMSO, pH 10.0) at 4°C. After lysis, the slides were washed three times in cold water and placed in a horizontal gel electrophoresis apparatus containing freshly prepared electrophoresis buffer (1 mM EDTA, 300 mM NaOH, pH = 13.0) for 20 min to allow DNA unwinding. Electrophoresis was then carried out at 20 V and at a starting current of 300 mA for 20 min at 4°C. Thereafter, the slides were neutralized with three washes of 0.4 M Tris, pH = 7.5, dehydrated with ethanol and dried. The slides were stained with 20 µg/ml ethidium bromide solution and viewed under a fluorescent illumination using 530–550 nm excitation filter and 590 nm barrier filter (U-MNG cube, Olympus, Germany). All steps were conducted in the dark to prevent additional DNA damage. To evaluate comet parameters, slides were examined in parallel using Viscomet image analysis software. A total of 150 randomly chosen cells from triplicate slides were examined for each sample (50 cells per slide). Image analysis was performed at ×200 magnification. The cell images were projected onto a high-resolution Heper-HAD™ (Sony, Japan) CCD camera (8 bits [Applitec, Israel, LIS-700]) and analyzed with Viscomet image analysis software using the MV Delta frame grabber (Matrix Vision, Germany). DNA damage was measured using the following parameters: comet extent (the distance from the leading head edge to the trailing edge of the tail), percentage tail DNA (percentage of DNA in tail), and tail extent moment (tail length X percentage tail DNA). Slides were coded and a single investigator analyzed all slides to minimize scoring variation.

### Migration Assays

Migration of cells was evaluated by two assays: the wound healing assay and a modified Boyden chamber assay (transwell assay).

The wound healing assay was performed according to ref [22]. Cells were seeded at a density of 0.2×10^6^ cells/well in six-well culture plates and allowed to form a confluent monolayer. The layer of cells was then scraped with a P200 pipette tip to create a wound of ∼1 mm width. Images of the wounds were captured at *t* = 0 and 24 h at ×40 magnification and the wound area was determined using the Scion Image for windows alpha 4.0.3.2 software. The ability of the cells to close the wound, that is, their motility, was evaluated by determining the healed area. Percentage of healed area was calculated in the following way: (wound area at *t* = 0−wound area at *t* = 22)/wound area at *t* = 0)×100. The plates were marked to ensure consistent photo-documentation.

The Modified Boyden chamber was performed as described previously [23]. Cells were seeded in triplicates on polycarbonate filters with 8 µm pores positioned on the upper compartment of 24-well transwell chambers (Costar, Cambridge, Mass, USA). The bottom surface of the transwell chambers was coated with fibronectin (5 µg/filter). Culture medium was added to the lower compartment. The cells were allowed to migrate for 18 hours at 37°C and the filters were then fixed with methanol and stained with Giemsa stain. The cells on the upper surface of the filter were removed with a cotton swab. Finally, cells that have migrated to the bottom side of the filter were photographed and counted under a microscope.

### Animals

Athymic female or male BALB/c *nu/nu* mice, 5–6 week old, were purchased from Harlan Ltd. (Jerusalem, Israel). The mice were housed under specific pathogen-free conditions in top filtered cages and fed a regular diet. All procedures were conducted using facilities and protocols approved by the Animal Care and Use Committee of the Hadassah-Hebrew University School of Medicine.

### Human Xenograft Murine Model

CRL 1867 cells (human pancreas carcinoma) were harvested by trypsinization of confluent cultures, washed, and resuspended at 1.0×10^8^ cells/ml in growth medium. Mice were inoculated with CRL 1687 cells in 100ul medium under the dorsal skin one day following whole body irradiation at 400 cGy, delivered by a linear accelerator (Varian Climac 6×) at a source to skin distance of 80 cm, and a dose rate of 170 cGy/min. Mice were treated with 30mg/kg i.p. GRN 163L 3 times per week for several weeks, starting one day after tumor injection. The control group was injected with PBS (0.2 ml i.p.). Tumor growth was monitored every 6–8 days by caliper measurements of two perpendicular diameters of xenografts (D_1_, the larger diameter and D_2_, the smaller diameter). Tumor volume was measured using the formula: π/6 (D_1_×D_2_), and expressed as mean tumor volume ± SE (in mm^3^). At 41–55 days after cell injection, at the time of killing, tumors were excised free of connective tissue, weighted and half of them were frozen in liquid nitrogen. The other half was used for telomere length determination and pathological parameters.

### Histology

At the end of the experiments, mice were sacrificed and the tumor, pancreas, and lung tissue were removed. Tissues were fixed in 10% formalin, embedded in paraffin, stained with hematoxylin and eosin, and evaluated by light microscopy.

## Results

### *In Vitro* Studies

#### Telomerase inhibition with GRN163 shortens telomeres

**Figure 1:**
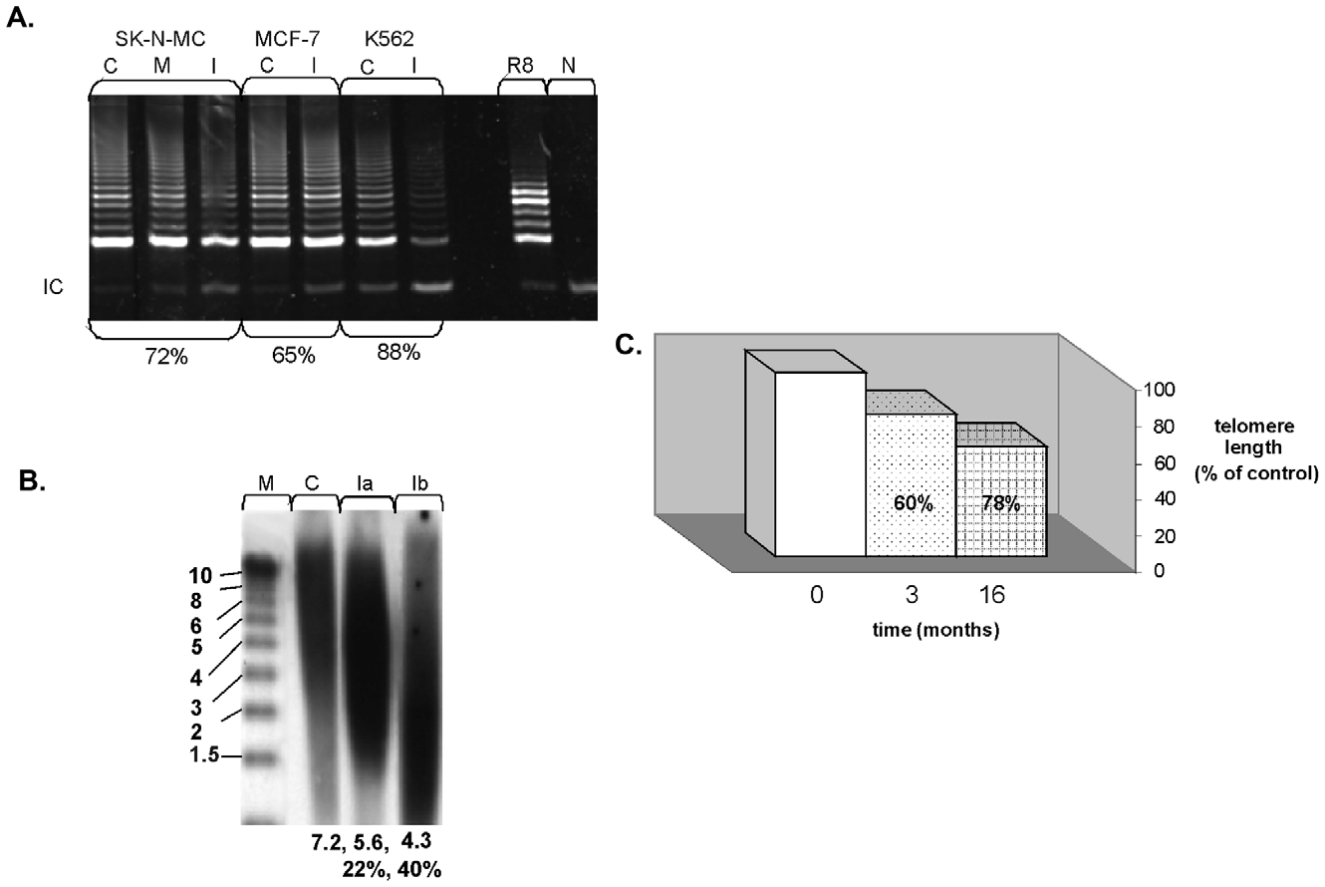
GRN163 inhibits telomerase activity in a sequence specific manner and shortens telomeres. a. SK-N-MC, MCF-7 and K562 were exposed to 5uM of GRN163. Telomerase activity was assessed by the TRAP assay after 24hours. C- control untreated cells, M- mismatch nonspecific scrambled oligo, I-telomerase inhibitor GRN163. R8- standard TRAP control, N- negative control with no cell extracts, IC- internal PCR control. The extent of telomerase inhibition is denoted in percentages below the lanes. b. SK-N-MC cells were continuously exposed to 5µM of GRN163. Telomere length was measured by Southern blot. C- control untreated cells, Ia- telomere length of cells exposed to telomerase inhibitor for three months, Ib- telomere length of cells exposed to telomerase inhibitor for 16 months, M- molecular size marker. Mean telomere length is denoted below each lane, and the percent of telomere shortening is shown as well. c. Graphical presentation of the extent of telomere shortening after the exposure of SK-N-MC cells to GRN163 for 1 year as measured by Southern blot.

Administration of GRN163 resulted in 70–90% inhibition of telomerase (Fig. 1a). This inhibition persisted up to 72 hours and repeated measurements throughout the 16 months of the experiment verified continuous inhibition of telomerase at this range (not shown). In all cell lines the telomeres were shortened in a range of 20–30% and 40% after 3 and 16 months, respectively (Fig. 1 b).

#### Telomere shortening but no telomerase inhibition *per se* sensitizes cells specifically to cisplatinum

**Figure 2:**
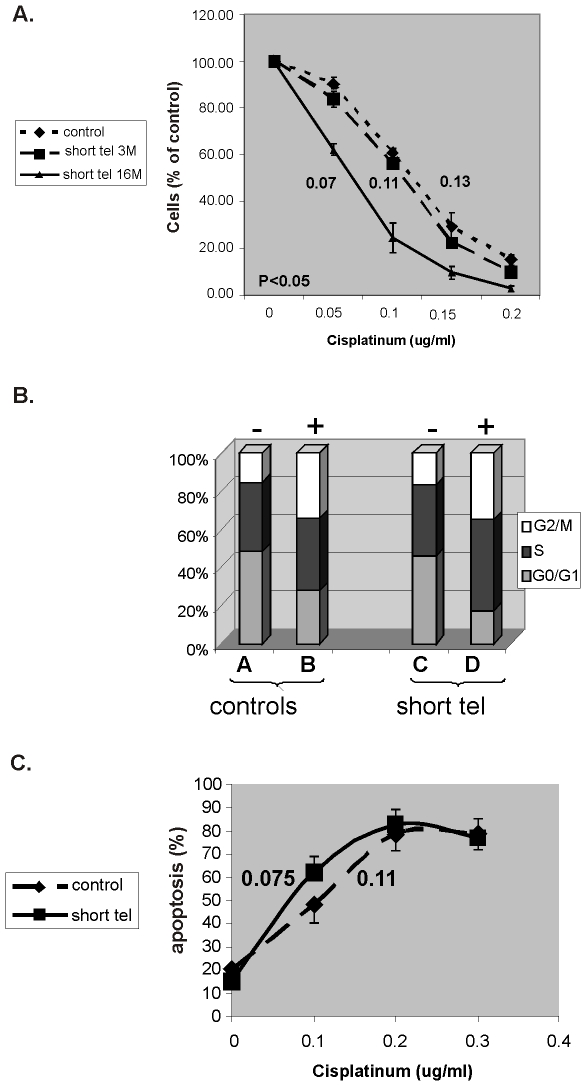
Sensitivity of SK-N-MC cells to cisplatinum is telomere length dependent. a. Proliferation of cells with shortened telomeres exposed to cisplatinum. SK-N-MC cells were continuously exposed to GRN163. The sensitivity of the cells to cisplatinum was estimated after three and 16 months of telomerase inhibition. Numbers indicate the IC_50_ of the drug in these time points (after 22% and 40% reduction in telomere length). P value refers to difference between WT and short tel-16 months. b. Cell cycle status of SK-N-MC cells with shortened telomeres exposed to 0.13µg/ml cisplatinum. SK-N-MC cells with shortened telomeres were exposed to cisplatinum and the cell cycle status was evaluated by FACS. + or − refers to the exposure to cisplatinum. C. The apoptotic index of SK-N-MC cells with shortened telomeres exposed to cisplatinum. The same cells were analyzed by FACS for their apoptotic index, as represented by their preG1 status. The numbers indicate the LD_50_ of cisplatinum in cells with intact or shortened telomeres.

As shown in Fig. 2 and Table 1 telomere shortening increased the sensitivity of the three cell lines to cisplatinum in a length dependent manner. Telomerase inhibition *per se* had no independent effect on cells sensitivity to cisplatinum. The IC_50_ of cisplatinum decreased from 0.13µg/ml to 0.07µg/ml after 16 months. Telomere shortening did not affect significantly the cells' sensitivity to doxorubicin and vincristine. Table 1 summarizes these results and Fig. 2 shows in details the changes in sensitivity of SK-N-MC cells to cisplatinum. Since all cell lines behaved similarly in this respect, we selected SK-N-MC cells as a model for further characterization of the mechanisms underlying the telomere shortening induced sensitization of cancer cells to chemotherapy and the differential sensitivity to cisplatinum.

**Table 1:** Effects of telomerase inhibition and telomere shortening on the sensitivity of cancer cells to cytotoxic drugs.

Telomere length		TA	SK-N-MC		
			IC_50_ of Vincristine (ng/ml)	IC_50_ of Doxorubicin ng/ml	IC_50_ of Cisplatinum ug/ml
Intact telomeres		+	8	49	0.13
		−	8	50	0.13
Shortened telomeres	∼75% of the original size	+	8.3	50	0.11
		−	8	49	0.11
	∼60% of the original size	+	7.9	49	0.07*
		−	7.85	49	0.07*

Telomere length		TA	K562		
			IC_50_ of Vincristine (ng/ml)	IC_50_ of Doxorubicin ng/ml	IC_50_ of Cisplatinum ug/ml
Intact telomeres		+	12	125	3.02
		−	12	125	3
Shortened telomeres	∼75% of the original size	+	13	125	2.1
		−	13	125	2.1
	∼60% of the original size	+	12	123	1.7*
		−	12.5	124	1.65*

Telomere length		TA	MCF-7		
			IC_50_ of Vincristine (ng/ml)	IC_50_ of Doxorubicin ng/ml	IC_50_ of Cisplatinum ug/ml
Intact telomeres		+	10	250	3
		−	10	250	3
Shortened telomeres	∼75% of the original size	+	10	250	2.5
		−	10	250	2.5
	∼60% of the original size	+	9.8	250	1.6*
		−	10	250	1.6*

*- significance of P<0.05.

TA- telomerase activity.

#### Telomere shortening does not affect the cell cycle status after the exposure to cytotoxic drugs

Telomere shortening did not affect the cell cycle status of the cells (bars A and C in Fig. 2b). Exposure of the cells to cisplatinum resulted in decrease of G0/G1 and increase of G2/M stages of the cell cycle. These changes were not affected by the shortening of telomeres (fig. 2b). Also, telomere shortening did increase the apoptotic rate of the cells due to chemotherapy (Fig. 2c).

#### Telomere shortening slower the migration of cancer cells

**Figure 3:**
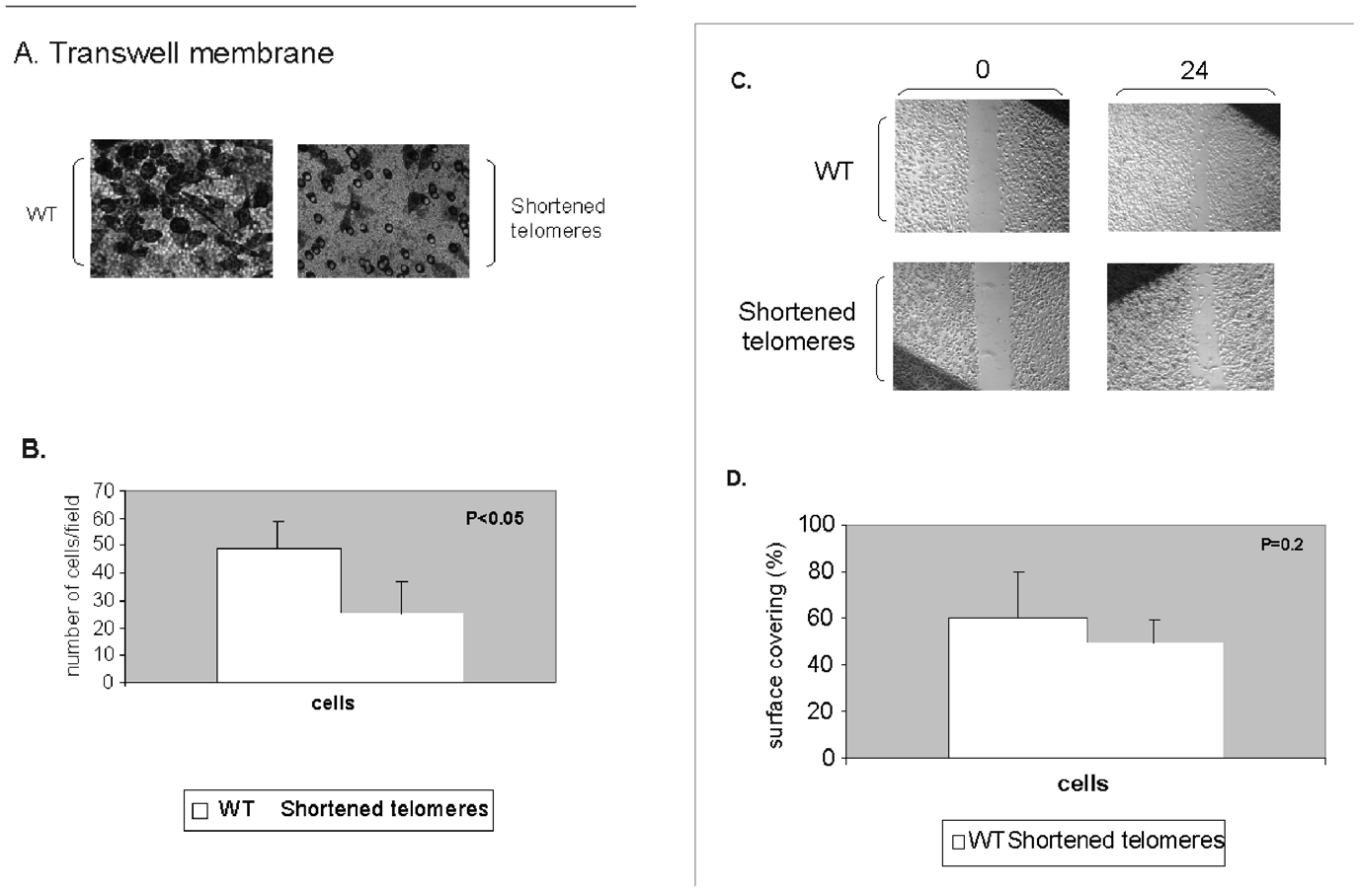
The migration ability of cells with shortened telomeres. The migration of cells with shortened telomeres was evaluated by the transwell and the wound healing assays. a. The transwell membrane assay. Cells with intact or shortened telomeres were allowed to migrate through a membrane for 16 hours, Gimza stained and counted. A representative picture is shown. b. Graphical demonstration of the average cell counts of four independent experiments. c. The wound healing assay. Cells with shortened telomeres were plated on Petri dishes, and the culture was “scratched” and followed for 24 hours. Average measurements of the cell free gaps were done. A representative example is shown. d. Graphical demonstration of the average cell counts of four independent experiments.

Since *in vitro* migration of cancer cells is considered a valid representation of their metastatic potential, we evaluated the effect of telomere shortening on this feature as well. The migration was assessed by transwell membrane (Boyden chamber) (Fig. 3a, b) and the wound healing (Fig. 3c, d) assays. As shown in Fig. 3, on the transwell membrane assay the cells with shortened telomeres migrated significantly slower than the control cells. The wound healing assay also demonstrated tendency towards slower migration, but the results did not reach statistical significance.

The subsequent studies were performed in order to elucidate the potential mechanisms underlying the phenotypic changes induced by telomere shortening.

#### Telomere shortening is associated with increased DNA damage and impaired DNA repair ability as evaluated by comet assay. Cisplatinum further impairs the DNA repair in cells with shortened telomeres

**Figure 4:**
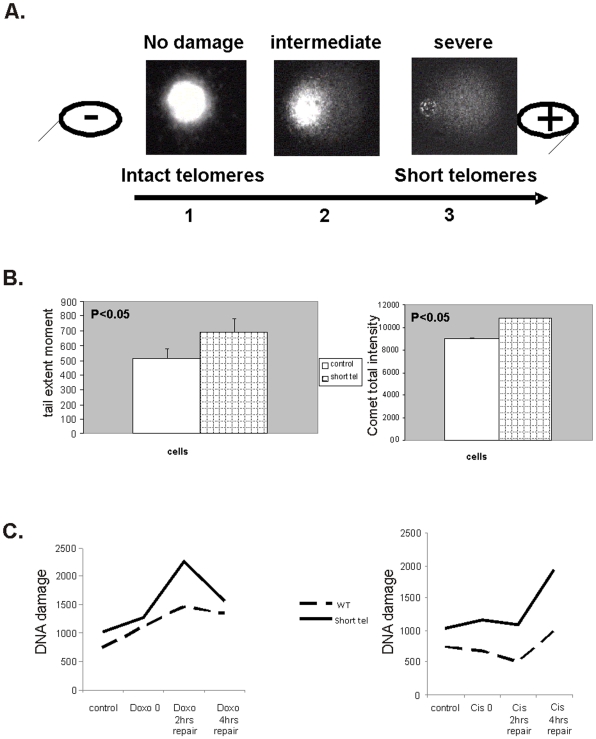
DNA damage status and repair ability of cells with shortened telomeres- the Comet assay. a. The principle of the comet assay. Cells nuclei were exposed to electrophoresis and stained with ethidium bromide. Broken DNA migrates out of the nuclei and forms the comet. Three degrees of DNA damage are shown: control nuclei with intact DNA (representing DNA damage in cells with intact telomeres, #1), intermediate state in cells with partially broken DNA (representing cells with mild telomere shortening, #2) and cells with shortened telomeres harboring damaged broken DNA (representing cells with shortened telomeres, #3). b. Quantitation of the extent of the DNA damage status of cells with shortened telomeres, performed by screening 50 images per sample in quadruples. On the left- Tail extent moment parameter, on the right- total comet intensities. c. Quantitation of the extent of the ability of the cells to repair DNA damage applied by doxorubicin (right panel) or cisplatinum (left panel) 2 and 4 hours post drug induced damage. The assay was performed by screening 50 images per sample in quadruples. The DNA damage status was determined by calculating the tail extent moment.

Telomeres shortening may enhance DNA damage response due to loss of its protective function. To evaluate the level of the DNA damage in cells with shorter telomeres, we employed the comet assay. As shown in Fig. 4a, b telomere shortening was associated with DNA damage in the cells. Tail extent moment parameter (reflecting tail length X percentage tail DNA, while tail length is the distance in µm from the head center to the end of the tail) was 30% higher in the cells with shorter telomeres. Assessment of all other comet assay parameters revealed similar results, and the overall comet intensity increased in about 16% in these cells.

To understand why cells with shorter telomeres are more sensitive to cisplatinum than to doxorubicin, the cells were exposed to these drugs for one hour followed by changing the medium to medium without drug. DNA damage levels were evaluated 2 and 4 hours post drug exposure using the comet assay. As shown in Fig. 4c, both drugs induced DNA damage, but the cells with shortened telomeres failed to repair the DNA damage caused by cisplatinum, while the doxorubicin induced damage was repaired in a similar manner by cells with both intact and shortened telomeres.

#### Telomere shortening is associated with formation of telomere damage induced foci (TIF) whose repair is impaired in cells with shortened telomeres. Cisplatinum further impairs the clearance of TIFs following telomere shortening

**Figure 5:**
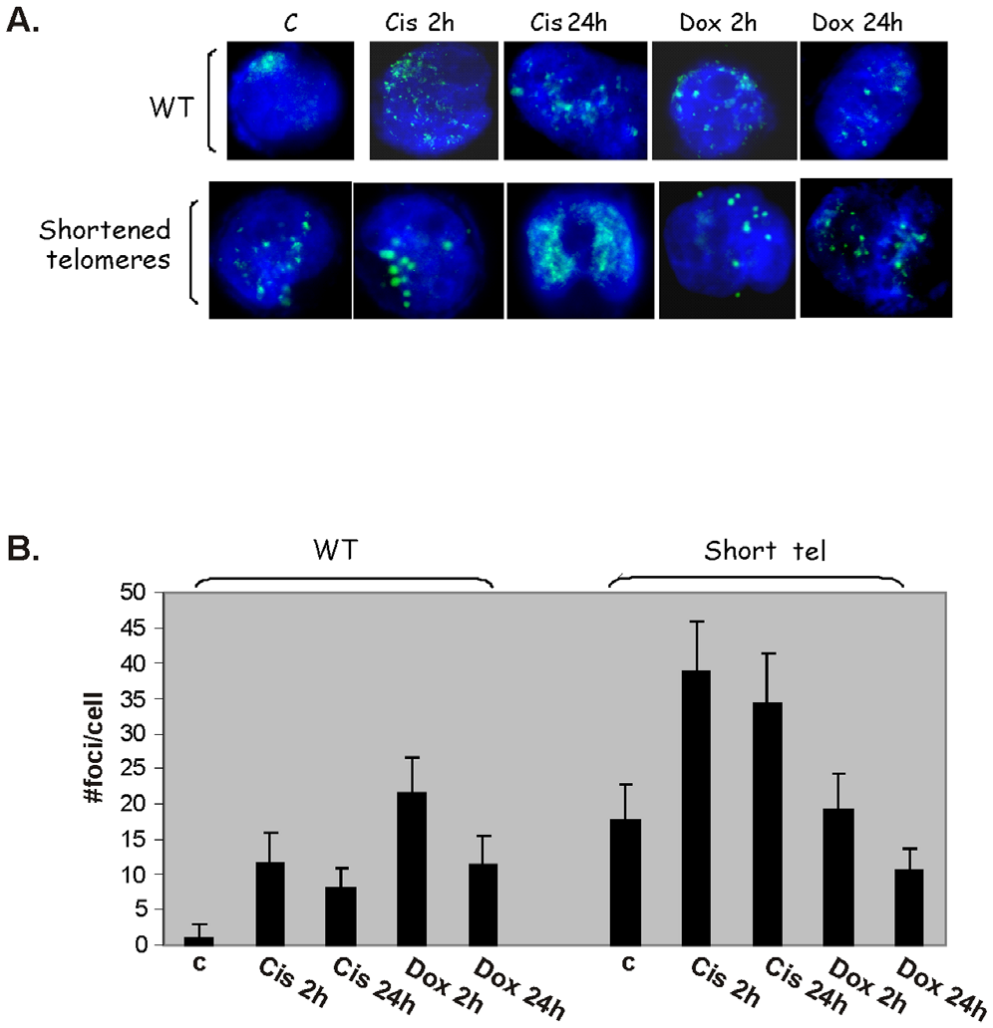
The formation of γH2AX foci in cells with shortened telomeres. SK-N-MC cells were exposed to cisplatinum or doxorubicin for 1 hour and then resuspended in drug free medium. The formation of γH2AX foci was followed by immunofluorescence at the indicated time points after the releasing from the drugs. Blue color indicates nuclei stained with DAPI, green foci were detected using cy2-labeled anti γH2AX antibody. A- A representative example. B- Quantitation of the number of foci per sample. Averages were calculated for at least 50 nuclei were counted for each duplicated sample and two experiments which yielded similar results.

DNA damage response at telomeres is manifested by appearance of TIF containing γH2AX. We followed the formation of γH2AX foci in cells with shortened telomeres and following treatment with cisplatinum or doxorubicin. Telomere shortening resulted in the appearance of TIF. Administration of cisplatinum or doxorubicin also caused TIF in cells with intact telomeres and increased their number in cells with shortened telomeres (Fig. 5a, b). We did not verify, however, whether these TIF were associated with telomeres only, or were spread throughout the genome.

TIF formation is an indicator of the DNA repair ability of the cells. To assess this property of cells with shortened telomeres, we followed the disappearance of TIF after withdrawal of the cytotoxic drugs. In cells with shortened telomeres exposed to cisplatinum the number of TIF did not change significantly up to 24h after the withdrawal of the drug from the growth medium (Fig. 5a, b). In contrast, the number of TIF induced by doxorubicin decreased by 50% after 24h in these cells, implying that the damage induced by this drug does not impair the DNA repair process in cells with shortened telomeres. Collectively, these data may explain the differential sensitivity of cells with shorter telomeres to cisplatinum, which probably stems from their inability to cope with DNA adducts induced by cisplatinum versus DNA double stranded breaks caused by doxorubicin.

#### Telomere shortening affects micro RNA expression

The above mentioned phenotypic changes caused by telomere shortening in cancer cells are probably caused by alterations in gene expression. We decided to look at the changes in miRNA expression as an initial step to study the genetic changes induced by telomere shortening. We performed profiling of expression of ∼900 micro RNA (miR) comparing SK-N-MC cells with intact telomeres (about 7kb), shortened telomeres (about 4 kb) and cells whose shortened telomeres were elongated back to their original size (about 7 kb) by reconstitution of telomerase activity. While cells with re-elongated telomeres exhibited similar pattern of miR expression compared to the cells with intact telomere length, cells with shortened telomeres demonstrated different profile of miR expression. The complete list of the up and down regulated miRs is beyond the scope of this paper and will be reported in another publication. Interestingly, some of the miRs with differential expression are related to cellular processes such as growth inhibition, apoptosis or malignant processes and may explain the sensitization to cisplatinum and impaired migration (Table 2). The table depicts several miRs that may have biological relevance to the characteristic phenotype of cells with shortened telomeres illustrated in our study. These differences in miR expression may serve as a starting point to explore the genetic changes caused by telomere shortening leading to the above described phenotype of cancer cells.

**Table 2:** miRs that were differentially expressed in cells with shorter telomeres.

miR symbol	Fold change (WT/short tel signal)	Putative relevance to the phenotype of cells with shorter telomeres
**Upregulated in cells with shortened telomeres**		
hsa-let-7a	0.152	Possess anti growth effects in cancer cells *in vitro* (lung cancer, melanoma) and *in vivo* (mouse models of lung cancer).
hsa-let-7b	0.0427	
hsa-let-7c	0.1509	
hsa-let-7d	0.1491	
hsa-miR-128	0.1571	May be involved in Alzheimer pathology, or in ageing process in general
hsa-miR-138	0.0793	Targets telomerase, is downregulated in squamous cell carcinoma of the tongue, anaplastic thyroid carcinoma and papillary thyroid carcinoma
hsa-miR-143	0.157	A tumor suppressor mir. Targets ERK5 and may potentially target kRas
hsa-miR-148a	0.1156	A tumor suppressor gene. Targets human DNMT3b protein, resulting in growth suppression.
hsa-miR-181a	0.1362	Targets oncogenes such as: the oncogenes 70-kD zeta-associated protein and Tcl1 in CLL
hsa-miR-181b	0.0709	
hsa-miR-193a-3p	0.0848	A pro apoptotic miR, activates the caspase cascade.
hsa-miR-199a-3p	0.0684	a pro-apoptotic agent, targets MET and ERK2 proto-oncogenes.
hsa-miR-199a-5p	0.0077	
**Upregulated in cells with intact telomeres**		
hsa-miR-125a-5p	3.8808	Was upregulated in serous ovarian carcinoma and neuroblastoma, implicated in proliferation and migration processes
hsa-miR-140-3p	3.5167	Is involved in cell growth in lung carcinoma cells, targets histone deacetylase 4 which binds 53bp1 and involved in DNA repair
hsa-miR-146b-5p	9.3262	Is associated with inflammatory processes, is induced by NF-kappa B.
hsa-miR-17	5.9754	miRs 17, 18a and 19a belong to the 17-92 cluster which is associated with processes related to proliferation and aggressiveness of malignancies and defined as pro-tumorigenic.
hsa-miR-18a	8.9537	
hsa-miR-19a	7.0316	
hsa-miR-192	3.0604	Its expression activates the survivin promoter in lung A549 cells, regulates cancer cell growth. Targets SIP1.
hsa-miR-21	11.5308	Targets sprouty2 and promotes cellular outgrowths and carcinogenesis. It targets tumor suppressor genes in invasion and metastasis.

### *In Vivo* Studies

#### Telomerase inhibition by GRN163L does not prevent implantation of CRL 1687 tumor but slows the tumor growth

**Figure 6:**
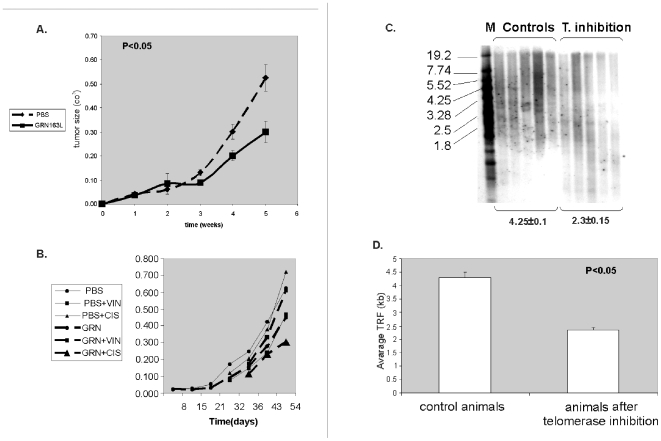
Tumor dimensions after the inhibition of telomerase. CRL 1687cells were injected subcutaneously to nude athymic mice, which were treated three times a week with telomerase inhibitor, GRN163L or with PBS as a control. a. Average tumor sizes of the two groups of mice (each contained 18 mice) which were measured every week. b. Each group of mice was subsequently divided into three subgroups: one treated with PBS or GRN163L only, the other treated with doxorubicin and PBS or GRN163L, and a group treated with vincristine and PBS or GRN163L. The sizes of xenografts were measure each week. c. *In vivo* telomere shortening after GRN163L administration. Nude athymic mice were injected with CRL1687 cells subcutaneously and treated with telomerase inhibitor, GRN163L, or PBS. Telomere length was evaluated by Southern blot. M- molecular size marker (sizes are listed on the left). Numbers below the gel indicate average telomere length in KB and the extent of telomere shortening. 5 samples of each cell type were analyzed. D. Graphical representation of telomere shortening *in vivo* after telomerase inhibition with GRN163L. TRF- terminal repeats fragment indicated the length of telomeres. The telomeres shortened by ∼40% after telomerase inhibition. The experiment was repeated twice with 5 samples of each cell types.

BALB/c nude mice were injected with CRL1687 cells (pancreatic adenocarcinoma) and treated with GRN163L, a telomerase inhibitor, three times a week (30mg/kgBW) as described previously [24]. We selected these specific cells for our studies due to their excellent response to telomerase inhibitor GRN163L (not shown), which was better than the response of 5 other cell line tested. Since telomere shortening induced drug sensitivities in several cell lines, we assumed that using another cancer cell line for the in vivo studies will not affect the results. Three days telomerase inhibition in these cells did not affect their implantation rate in the animals compared to cells with active telomerase (not shown). Treatment with the telomerase inhibitor resulted in decreased growth rate of the tumors (Fig. 6a). After 5 weeks the average tumor size in the control group, treated with PBS, was 0.526±0.055 cm^3^, whereas the in the GRN163L treated mice the tumors dimensions were 0.299±0.045, cm^3^ a statistically significant difference.

#### The combined effect of cisplatinum or vincristine on xenograft sizes with inhibited activity of telomerase

To verify the *in vitro* results regarding the combined effect of telomerase inhibition and chemotherapy, mice were treated with either vincristine or cisplatinum after 5–7 weeks of telomerase inhibition. Since tumor sizes varied between the groups due to the inhibition of telomerase, we started the treatment after reaching similar size of tumors at both groups. Accordingly, the control (PBS treated) group was exposed to chemotherapy after 5 weeks and the telomerase inhibited group was exposed to the drugs after 7 weeks. 0.4mg/kgBW vincristine was injected i.p. once a week every week for 7 weeks and 3 mg/kgBW cisplatinum was applied i.p. twice a week every two weeks. These concentrations were calibrated prior to the experiment and were the highest doses that did not harm the animals' weight or their general well being (not shown). Animals were sacrificed 7 weeks after the completion of each treatment and tumor size was evaluated (Fig. 6b). Tumors exposed to telomerase inhibitors were smaller than their control counterparts (weight: 0.49±0.31 vs. 0.58±0.16 gr′ respectively, size: 0.33±0.24 vs. 0.64±0.09 cm^3^ respectively) with cisplatinum being synergistic with telomere shortening. Telomere shortening did not increase the efficacy of vincristine in terms of tumor growth.

The effect of telomerase inhibition on telomere length in the animals was evaluated after 5 weeks of administration of GRN163L. Mice were sacrificed and the average length of their tumor telomeres was evaluated by Southern blotting (Fig. 6c). In all tumors, the average telomere length decreased by 50% (from 4.25 kb to 2.3kb). Interestingly, the *in vivo* telomere shortening was much more rapid than that *in vitro*. The *in vitro* inhibition for 16 months achieved 40% telomere reduction while *in vivo*, 50% reduction was obtained after 5 weeks only. Since the mice telomeres were much longer (∼50kb) from that of the tumor cells (∼4 kb), they did not interfere with the interpretation of the results regarding telomere length of the treated tumors *in vivo*. Fig. 6 c shows only the CRL1867 telomeres, while the mice telomeres are not shown.

#### Histological parameters of xenografts from mice treated with telomerase inhibitor and cytotoxic drugs

**Figure 7:**
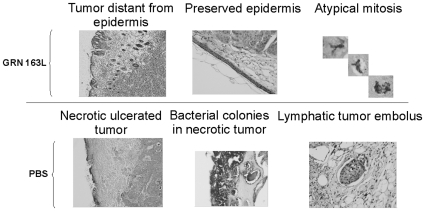
Histological analyses of tumors treated *in vivo* with telomerase inhibitor. Tumors grown in athymic nude mice treated with telomerase inhibitor GRN163L were analyzed histologically after Gimza staining. The upper panel demonstrates less aggressive parameters of tumors treated with telomerase inhibitor, and the lower panel show a more aggressive appearance of tumors treated with PBS only.

Telomere shortening affected also the histological appearance of the tumor. The GRN163L treated tumor exhibited less tumor lymphatic emboli, less foci of necrosis and tumor distance from epidermis as the main features. All these findings suggest that telomere shortening causes the tumor to acquire a less aggressive phenotype. These histological findings are summarized in Table 3 and shown in Fig. 7. The histological effects of cisplatinum and vincristine administration were not as affected as tumor kinetics by telomere shortening, probably due to the considerable tissue damage exerted by the cytotoxic drugs *per se*.

**Table 3:** Histological analyses of tumors with shortened telomeres.

Overall impression	Invasion to lymphatic vessels	Atypical mitoses	Invasion to epidermis	Ischemic necrosis and ulcer	treatment
Very aggressive	++	++	++	++	PBS
Non aggressive	−	−	−	−	GRN163L

• ++ denotes high frequent distribution of the described feature.

• − denotes no detection of the described feature.

## Discussion

In this study we systematically addressed several questions concerning the effect and mechanism of perturbations of the telomere/telomerase system as a therapeutic modality in cancer cells. The results of the study show that the most important goal in this regard is significant telomere shortening. In spite of other roles of telomerase in the cell it seems that chemosensitization of cancer cells requires telomere shortening and telomerase inhibition is a mean to achieve this end point. The features of cells with shortened telomeres described in our paper were obtained only after a significant (∼40%) attrition of telomeres. This conclusion is supported also by results of other studies using different approaches and addressing different questions, all related to the effect of telomere/telomerase perturbation on the cancer cell. For example, shortening of telomeres by targeting telomerase using various strategies caused inhibition of cancer cells growth [25]. Cells isolated from mTERC^−/−^ mice with shortened telomeres were more sensitive to double strand breaks forming drugs [10]. Likewise telomerase inhibition by small-molecule (BIBR1532) sensitized drug-resistant and drug-sensitive cells to chemotherapeutic treatment in a telomere length dependent manner [26]. Similarly, shortening of telomeres caused by siRNA to telomerase sensitized cancer cells to ionizing radiation and chemotherapy [27]. Additionally, telomerase inhibition enhanced the response to anticancer drug treatment in human breast cancer cells [28]. Other studies have shown a similar effect on various cancer cell lines [29]–[31]. Another report pointed to telomere structure rather than its length as a cause of increased susceptibility of tumor cells to anticancer drugs [32]. Several other studies, however, reached different conclusions by showing that telomerase inhibition sensitized cancer cells to cytotoxic treatment independently of telomere shortening [33]–[35]. This was attributed to a capping protecting function of telomerase on telomeres. The contribution of our study is the systematic approach focusing on the effect of telomere shortening versus telomerase inhibition. By creating an experimental system which included cells with inhibited versus active telomerase (with shortened and intact telomeres) we were able to show that telomerase inhibition *per se* does not confer sensitivity to chemotherapy in our experimental setting.

For the initial characterization of cells with shortened telomeres we have used selected representatives of malignant cells: mesenchymal cells (SK-N-MC), hematological malignant cells (K-562) and epithelial cells (MCF-7). All cells reacted similarly in terms of drug sensitivity after telomere shortening; therefore we speculated that the sensitivity of cells with shortened telomeres can be regarded a general phenomenon common to the majority of malignant cells. Therefore we continued the further studies on one cell line only.

This study demonstrates that the synergism between telomere shortening and exposure to cytotoxic drugs is related to the mechanism of action of these drugs. Telomere shortening resulted in increased sensitivity only to cisplatinum, while the IC_50_ of doxorubicin and vincristine did not change. We obtained similar results in three different types of cancer cells. However, we cannot conclude that this is a general phenomenon, since other groups reported that telomere shortening sensitisized normal and neoplastic cells derived from telomerase RNA null mice to doxorubicin [10]. Another study showed that shortening of telomeres in breast cancer cells sensitized these cells to cytotoxic drugs regardless of their mechanism of action [28]. These differing results may stem from different biological characteristics such as damage response pathways in various tumor cells, which according to the results of this study, may be the main venue of chemosensitization [36].

The particular association between telomere/telomerase and cisplatinum is not surprising in view of the reported effects of cisplatinum on this system [37]. Cisplatinum has been shown to associate specifically with the G-rich telomere strand [37] and also inhibited telomerase activity in numerous types of cancer cells [e.g. ref 38]. These findings, however, do not explain the mechanism of sensitization demonstrated in our study. Therefore, we attempted to explore the possible mechanisms of this differential sensitization. The results point to impairment of specific types of DNA damage repair that may be affected by telomere shortening. As there are no apparent common structural features among doxorubicin mediated DNA lesions and the 1,2- and 1,3-diguanyl DNA intrastrand crosslinks, which are the major cisplatinum DNA adducts [39], their repair could be exerted by different mechanisms. Doxorubicin induces single- and double-strand breaks in DNA resulting from its binding to the DNA and blocking its unwinding and helicase activities, leading to inhibition of replication and transcription. This damage is repaired mainly by homologous recombination via the mismatch repair pathway through a mechanism distinct from the manner by which covalent DNA lesions produced by cisplatinum are processed [40]. This pathway is probably intact or less affected in telomere shortened phenotype. In contrast, DNA repair of single strand damage formed by cisplatinum exerted by the general nucleotide excision repair pathway, which differs from the mismatch repair pathway components and mechanism of action [41], is probably damaged or slowed with telomere shortening. These results may explain also the fact that the effect of vincristine, which does not damage DNA directly, is not affected at all by the shortening of telomeres. The findings of this study suggest that DNA damage triggered by telomere shortening is accentuated further by administration of cisplatinum and doxorubicin. However, whereas doxorubicin damage is repaired relatively well both in wild type and shortened telomeres, the DNA damage repair after cisplatinum is impaired by telomere shortening. The precise mechanisms of this phenomenon should be further explored.

An interesting connection between the ability of cells to repair DNA adducts and cellular or organism age was recently reported [42]. Incubation of mononuclear cells with cisplatinum induced DNA damage which were repaired faster in younger people compared to older ones [42]. As severe telomere shortening is one of the main mechanisms associated with senescence, it is reasonable to assume that cancer cells with shortened telomeres may have acquired several aspects of aged cells, among them the inability to repair DNA damage induced by cisplatinum.

These *in vitro* findings were demonstrated also in *in vivo* setting. Telomere shortening slowed the growth rate of tumor xenografts and verified the sensitization of the cells to cisplatinum. Moreover, telomerase inhibition affected not only the growth kinetics of the tumor, but also the biological characteristics as exemplified by the pathological evaluation. The histological findings showed that shortening of telomeres leads to a less aggressive phenotype of the tumor. These results are in concert with studies of Dikmen and Gryaznov [43] which reported similar histological finding after telomerase inhibition. The animal model part of our study revealed two other interesting findings. First, telomerase activity is not essential to the implantation of the tumor. It is important, though, to its further propagation which may stem from the fact that at the time of tumor inoculation the telomere length was still unaffected. This finding is in accordance with other studies, suggesting that initial tumor formation can occur in the absence of telomerase. Telomerase activity, however, is essential for tumor maintenance [44]. Second, *in vivo* telomerase inhibition results in much more rapid telomere shortening than that achieved *in vitro*. This finding may have clinical implications in future human studies of telomerase inhibitors.

In addition to chemosensitization, telomere shortening affected another feature of the cancer cell; its migratory potential. The migration assay is considered to reflect the metastatic potential of the cell. These results are in accordance with the report showing that telomere shortening resulting from rybozyme mediated suppression of mouse mTR significantly reduced the invasiveness and the metastatic potential of melanoma cells [45]. The same effect was demonstrated by another study in which hTERT-targeted RNA interference inhibited the tumorigenicity and motility of HCT116 cells [46].

It is plausible that the above mentioned findings are caused by genomic alterations triggered by telomere shortening. As an initial step towards characterization of these changes we evaluated the miRNA profile of the native versus telomere shortened cells. To our best knowledge, this approach has not been reported yet in a similar experimental setting. The profiling yielded distinct changes in expression of several miRNA. Some of them, which were upregulated in cells with shortened telomeres are more obvious candidates, such as miR-199a, miR-Let7a-d that are related to apoptotic or growth suppression processes (e.g. ref [47], and [48] respectively). Additionally, oncogene suppressors such as miR-181a,b [49], miR-148 [50], miR-143 [51], were highly expressed in these cells as well. miRs that are related to growth and proliferation were upregulated in cells with intact telomere length, including miR-21 [52], miR-146b-5p [53], miR-18a, miR-19a and miR-17, belonging to the 17-92 miR cluster [54], and miR-125a [55]. Additional data from our laboratory (not shown) found highly significant (p = 0.0014) overlap between some of these miR targets and proteins associated with sensitivity to cisplatinum. We are currently in the process of further studies attempting to determine the miRNAs and proteins with the most significant functions contributing to the biological phenotype of “shortened telomere” cancer cell.

Several studies found different miR expression in various experimental systems which confer resistance or sensitivity to cisplatinum. For example, exposure of ovarian cancer cells to cisplatinum resulted in the upregulation of miR-23b, miR-381, miR-340, and the downregulation of miR-520, miR-331, miR-185 and miR-106a [56]. The targets of these miRs overlapped with known proteins involved in cisplatinum resistance [56]. In MCF-7 cells, upregulation of miR-21 (which was upregulated in our shortened telomere cells) following dicer knockdown enhanced sensitivity to cisplatinum [57]. Mir-372 and miR-373 were reported as relevant to the response to cisplatinum in human testis cancer cell lines [58]. Let-7i (a member of Let 7 family of miRs that were upregulated in cells with shortened telomeres) was reported to significantly increase the resistance of ovarian and breast cancer cells to cisplatinum [59]. Another study demonstrated that let-7e, miR-30c, miR-125b, miR-130a and miR-335 were always diversely expressed in ovarian cancer cells resistant to cisplatinum [60]. Of these, the expression of miR-125b changed significantly in our system. In addition, miR-214 induced cell survival and cisplatinum resistance in ovarian cell carcinoma [61]. miR-98 was implicated in conferring resistance to cisplatinum in head and neck squamous cell carcinoma [62]. The diversity of this data suggests that miR signature can contain common as well as different components depending on the cell line and experimental conditions used.

The results of this study are relevant to the SKNMC cells, as other cancer cells may present different features. However, the similar effects of telomere shortening in three different cell lines suggest that the results of our study may be relevant to a wide array of cancer types.

These findings contribute to our knowledge concerning the optimal conditions for interventions in telomere/telomerase axis combined with conventional chemotherapy. Characterization of such conditions is important for establishment of future therapeutic schedules.

In summary, in this study we partially characterized the conditions and mechanisms by which telomerase inhibition affects cancer cells. Future studies will be focused at the molecular and genetic changes caused by telomere shortening. Inducing these changes will enable us to produce the “vulnerable” phenotype without the long lag period required for telomere shortening caused by telomerase inhibition.
